# Supplementary material for: Adolescents with a Favorable Mediterranean-Style-Based Pattern Show Higher Cognitive and Academic Achievement: A Cluster Analysis—The Cogni-Action Project
Source: Nutrients. 2024 Feb 23;16(5):608. doi: 10.3390/nu16050608 (PMC10934130; doi:10.3390/nu16050608)
Supplement: Supplementary file 1 [file nutrients-16-00608-s001.zip › nutrients-2803907-supplementary.pdf]

**Table S1.**	A	B	C	D	E	F	G	H	I	J	K	L	M	N	O	P	Q	R	S	T	U	V	W	X
A) Total cognitive perf	-																							
B) Cognitive flexibility	0.646 ***	-																						
C) Working memory	O.510 ***	0.000	-																					
D) Inhibitory control	0.465 ***	-0.000	0.000	-																				
E) Fluid reasoning	0.325 ***	0.000	-0.000	-0.000	-																			
F) Language	0.334 ***	0.210 ***	0.103 ***	0.230 ***	0.128 ***	-																		
G) English	0.366 ***	0.277 ***	0.077 **	0.225 ***	0.139 ***	0.594 ***	-																	
H) Mathematcs	0.388 ***	0.216 ***	0.168 ***	0.285 ***	0.105 ***	0.660 ***	0.532 ***	-																
I) Science	0.337 ***	0.189 ***	0.104 ***	0.239 ***	0.163 ***	0.655 ***	0.597 ***	0.688 ***	-															
J) History	0.366 ***	0.236 ***	0.126 ***	0.231 ***	0.138 ***	0.082 ***	0.523 ***	0.634 ***	0.637 ***	-														
K) Junkfood	-0.188 ***	-0.110 ***	-0.099 ***	-0.102 ***	-0.059 *	-0.100 ***	-0.089 **	-0.072 *	-0.083 **	-0.075 **	-													
L) Legumes	0.022	-0.038	0.054	0.037	0.004	-0.000	-0.022	0.020	-0.002	0.030	-0.031	-												
M) Pasta	-0.095 ***	-0.051	-0.043	-0.078 **	-0.009	-0.131 ***	-0.144 ***	-0.133 *	-0.129 ***	-0.124 ***	0.083 **	-0.036	-											
N) Gran	-0.047	-0.033	-0.009	-0.029	-0.024	‘-056 *	-0.023	-0.044	-0.042	-0.034	0.016	0.113 ***	0.028	-										
O) Olive oil	0.048	0.014	-0.001	0.032	0.077 *	0.108 ***	0.003	0.049	0.024	0.063 *	0.037	0.129 ***	-0.009	0.080 **	-									

P) Skip breakfast	-0.136 ***	-0.115 ***	0.002	-0.113 ***	-0.033	-0.091	-0.133 ***	-0.111 ***	-0.094 ***	-0.104 ***	0.137 ***	-0.031	0.103 ***	-0.101 ***	-0.047	-								
Q) Dairy breakfast	0.005	0.006	-0.026	0.022	0.015	-0.009	-0.014	-0.015	0.028	0.033	-0.012	0.094 ***	0.025	0.174 ***	0.108 ***	-0.160 ***	-							
R) Pastry breakfast	-0.182 ***	-0.075 **	-0.126 ***	-0.085 **	-0.088 **	-0.086 **	-0.071 *	-0.052	-0.050	-0.115 ***	-0.270 ***	-0.023	0.112 ***	0.080 **	0.050	0.104 ***	0.015	-						
S) Yogurt, cheese	-0.041	-0.051	-0.017	0.007	-0.003	-0.004	-0.029	0.013	-0.022	0.015	0.108 ***	0.104 ***	0.068 *	0.100 ***	0.039	-0.004	0.180 ***	0.095 ***	-					
T) Sweet	-0.214 ***	-0.145 ***	-0.095 ***	-0.130 ***	-0.037	-0.106 ***	-0.128 ***	-0.099 ***	-0.095 ***	-0.152 ***	0.232 ***	-0.109 ***	0.133 ***	-0.051	-0.045	0.213 ***	-0.037	0.294 ***	0.048	-				
U) Nuts	0.105 ***	0.067 *	0.067 *	0.041	0.025	0.020	0.040	-0.005	0.027	-0.001	-0.104 ***	-0.042	-0.026	-0.076 **	-0.074 **	-0.062 *	-0.031	-0.108 ***	-0.177 ***	-0.064 *	-			
V) Fruits	0.118 ***	0.081 **	0.054	0.055 *	0.039	0.038	0.105 ***	0.043	0.087 **	0.064 *	-0.062 *	-0.061 *	-0.058 *	-0.044	-0.115 ***	-0.011	-0.049	-0.059 *	-0.162 ***	-0.033	0.221 ***	-		
W) Vegetable	0.170 ***	0.110 ***	0.112 ***	0.052	0.055 *	0.058 *	0.084 **	0.055 *	0.045	0.066 *	-0.116 ***	-0.066 *	-0.018	-0.063 *	-0.067 *	-0.018	-0.095 ***	-0.145 ***	-0.148 ***	-0.117 ***	0.177 ***	0.346 ***	-	
X) Fish	0.081 **	0.035	0.066 *	0.079 **	-0.036	0.012	0.016	-0.008	-0.016	0.032	-0.137 ***	-0.026	-0.024	-0.043	-0.067 *	-0.043	-0.056 *	-0.140 ***	-0.118 ***	-0.097 ***	0.235 ***	0.113 ***	0.124 ***	-

Table S1. Correlation Matrix between indicators of cognitive and academic achievement and KIDMED indicators. Pearson's correlation coefficient *<0.05, **<0.01, ***<0.001.

		WD vs. LFV-HSD			WD vs. LFV-LSD			WD vs. MD			LFV-HSD vs LFV-LSD			LFV-HSD vs MD			LFV-LSD vs MD		
	**Analysis**	Diff.	t	p Bonf	Diff	t	p Bonf	Diff	t	p Bonf	Diff.	t	p Bonf	Diff	t	p Bonf	Diff	t	p Bonf
Fluid Reasoning	MM	-0.2885	-2.050	0.243	-0.2672	-1.924	0.328	-0.4063	-2.838	0.028	0.0212	0.317	1.000	-0.1178	-1.566	0.705	-0.1391	-2.007	0.270
	ANC 1	-0.2731	-1.043	0.314	-0.2424	-1.752	0.480	-0.3912	-2.7306	0.038	0.0307	0.460	1.000	-0.1182	-1.574	0.694	-0.1488	-2.159	0.186
	ANC 2	-0.29991	-2.136	0.197	-0.29237	-2.134	0.198	-0.43046	-3.099	0.012	-0.00753	0.114	1.000	-0.13955	-1.869	0.371	-0.14708	-2.129	0.201
SG Language	MM	-0.1833	-1.672	0.568	-0.3302	-3.042	0.014	-0.3840	-3.439	0.004	-0.1469	-2.813	0.030	-0.2007	-3.425	0.004	-0.0538	-0.996	1.000
	ANC 1	-0.1868	-1.708	0.528	-0.3346	-3.107	0.012	-0.3831	-3.443	0.004	-0.1477	-2.842	0.027	-0.1963	-3.361	0.005	-0.0486	-0.905	1.000
	ANC 2	-0.1910	-1.752	0.480	-0.3424	-3.218	0.008	-0.3907	-3.547	0.002	-0.1514	-2.945	0.020	-0.1997	-3.444	0.004	-0.0483	-0.900	1.000
SG Mathematics	MM	-0.1325	-0.984	1.000	-0.3001	-2.242	0.151	-0.3582	-2.611	0.055	-0.1676	-2.608	0.056	-0.2256	-3.143	0.010	-0.0581	-0.875	1.000
	ANC 1	-0.1209	-0.903	1.000	-0.2786	-2.114	0.208	-0.3379	-2.481	0.079	-0.1577	-2.481	0.079	-0.2170	-3.036	0.015	-0.0593	-0.903	1.000
	ANC 2	-0.1327	-0.995	1.000	-0.3007	-2.308	0.127	-0.3591	-2.664	0.047	-0.1679	-2.669	0.046	-0.2264	3.190	0.009	-0.0585	-0.891	1.000
SG History	MM	-0.0472	-0.426	1.000	-0.2259	-2.060	0.237	-0.2995	-2.651	0.049	-0.1786	-3.383	0.004	-0.2522	-4.251	<.001	-0.0736	-1.347	0.884
	ANC 1	-0.0443	-0.400	1.000	-0.2190	-2.009	0.268	-0.2895	-2.570	0.062	-0.1747	-3.321	0.006	-0.2451	-4.145	<.001	-0.0704	-1.296	1.000
	ANC 2	-0.0680	-0.615	1.000	-0.2632	-2.437	0.090	-0.3321	-2.970	0.018	-0.1951	-3.741	0.001	-0.2640	-4.486	<.001	-0.0689	-1.265	1.000
PISA	MM	-0.1905	-1.79	0.438	-0.3230	-3.06	0.013	-0.3914	-3.62	0.002	-0.1325	-2.62	0.054	-0.2009	-3.55	0.002	-0.0684	-1.31	1.000
	ANC 1	-0.1826	-1.73	0.504	-0.3089	-2.97	0.018	-0.3790	-3.53	0.003	-0.1263	-2.52	0.072	-0.1963	-3.48	0.002	-0.0701	-1.35	1.000
	ANC 2	-0.1904	-1.81	0.425	-0.3233	-3.15	0.010	-0.3929	-3.69	0.001	-0.1329	-2.68	0.045	-0.2025	-3.62	0.002	-0.0696	-1.34	1.000

Table S2. Sensitivity analysis with and without cluster. MM: Mixed Model. ANC 1: ANCOVA Analysis with “school type”. ANC 2: ANCOVA Analysis without “school type”

Table S3 shows the result of the latent class analysis with the number of participants for each item composing the clusters. These groups' differences are characterized by specific components which are represented by a unique profile found in the analysis.

	WD (1)		LFV-HSD (2)		LFV-LSD (3)		MED-DIET (4)	
**Indicator**	non-meet / meet	Group comp.	non-meet / meet	Group comp.	non-meet / meet	Group comp.	non-meet / meet	Group comp.
Fish	52/4	2,3,4	127/238	1,4	168/379	1,4	55/273	1,2,3
Second vegetable	55/1	2,3,4	283/82	1,3,4	377/170	1,2,4	7/321	1,2,3
Second fruit	55/1	2,3.4	242/123	1,3,4	466/81	1,2,4	10/318	1,2,3
Nuts	56/0	2,3,4	160/205	1,4	253/294	1,4	59/269	1,2,3
Junk Food	3/53	2,3,4	229/136	1,3,4	482/65	1,2	278/50	1,2
Pasta	0/56	2,3,4	73/292	1,3	225/322	1,2,4	94/234	1,3
Skip breakfast	14/42	2,3,4	250/115	1,3	492/55	1,2,4	240/88	1,3
Pastry breakfast	2/54	2,3,4	189/176	1,3,4	536/11	1,2,4	301/27	1,2,3
Sweet	6/50	2,3,4	120/245	1,3,4	547/0	1,2,4	265/63	1,2,3

Clusters composition.

*The number on the left side corresponds to the participants who do not meet the criteria evaluated as an indicator, while the number on the right side represents the participants who do respond to the criteria evaluated. 1: Western diet; 2: Low-fruit and vegetable and high-sugar diet; 3: Low-fruit and vegetable, low-sugar diet; 4: Mediterranean diet. Non-meet/meet the dietary indicator. Group comp: comparison between groups representing significant difference among them (p <0.05)

				95% Confidence interval				
**Domain**	**Status**	**Mean**	**SE**	**Lower**	**Upper**	**Comparisons**		***P-values***
Total Cognitive	WD	94.6	1.82	89.8	99.3	WD	LFV-HSD	0.015
	LFV-HSD	98.1	1.51	92.3	104.0	WD	LFV-LSD	<.001
	LFV-NSD	101.2	1.49	95.1	107.2	WD	MED-DIET	<.001
	MED-DIET	102.2	1.51	96.4	108.0	LFV-HSD	LFV-LSD	<.001
	-	-	-	-	-	LFV-HSD	MED-DIET	<.001
	-	-	-	-	-	LFV-LSD	MED-DIET	0.374
Cognitive Flexibility	WD	-0.4099	0.1522	-0.724	-0.0961	WD	LFV-HSD	0.317
	LFV-HSD	-0.1359	0.0913	-0.415	0.1427	WD	LFV-LSD	0.002
	LFV-NSD	0.0865	0.0862	-0.215	0.3877	WD	MED-DIET	0.001
	MED-DIET	0.1230	0.0927	-0.151	0.3970	LFV-HSD	LFV-LSD	0.006
	-	-	-	-	-	LFV-HSD	MED-DIET	0.004
	-	-	-	-	-	LFV-LSD	MED-DIET	1.000
Inhibitory Control	WD	-0.2263	0.193	-0.693	0.241	WD	LFV-HSD	1.000
	LFV-HSD	-0.1095	0.151	-0.675	0.456	WD	LFV-LSD	0.077
	LFV-NSD	0.1158	0.148	-0.477	0.708	WD	MED-DIET	0.142
	MED-DIET	0.0942	0.151	-0.465	0.654	LFV-HSD	LFV-LSD	0.004
	-	-	-	-	-	LFV-HSD	MED-DIET	0.037
	-	-	-	-	-	LFV-LSD	MED-DIET	1.000
Working Memory	WD	-0.3115	0.1511	-0.6218	0.00114	WD	LFV-HSD	1.000
	LFV-HSD	-0.1565	0.0901	-0.4238	0.11070	WD	LFV-LSD	0.066
	LFV-NSD	0.0426	0.0849	-0.2442	0.32933	WD	MED-DIET	0.005
	MED-DIET	0.1687	0.0915	-0.0946	0.43194	LFV-HSD	LFV-LSD	0.018
	-	-	-	-	-	LFV-HSD	MED-DIET	<.001
	-	-	-	-	-	LFV-LSD	MED-DIET	0.417

Fluid Reasoning	WD	-0.28438	0.1431	-0.5725	0.00371	WD	LFV-HSD	0.243
	LFV-HSD	-0.00410	0.0765	-0.2086	0.21677	WD	LFV-LSD	0.328
	LFV-NSD	-0.01715	0.0704	-0.2465	0.21225	WD	MED-DIET	0.028
	MED-DIET	0.12191	0.0782	-0.0875	0.33141	LFV-HSD	LFV-LSD	1.000
	-	-	-	-	-	LFV-HSD	MED-DIET	0.705
	-	-	-	-	-	LFV-LSD	MED-DIET	0.270

Table S4. Post Hoc results of mixed model in comparisons of Mediterranean-style based dietary patterns and cognitive domains.

				95% Confidence interval				
School Subject	Status	Mean	SE	Lower	Upper	Comparisons		*P-values*
Language	WD	5.11	0.1043	4.90	5.32	WD	LFV-HSD	0.568
	LFV-HSD	5.29	0.0457	5.19	5.40	WD	LFV-LSD	0.014
	LFV-NSD	5.44	0.0394	5.34	5.54	WD	MED-DIET	0.004
	MED-DIET	5.49	0.0476	5.39	5.60	LFV-HSD	LFV-LSD	0.030
	-	-	-	-	-	LFV-HSD	MED-DIET	0.004
	-	-	-	-	-	LFV-LSD	MED-DIET	1.000
English	WD	5.13	0.186	4.65	5.61	WD	LFV-HSD	0.009
	LFV-HSD	5.52	0.153	4.94	6.10	WD	LFV-LSD	<.001
	LFV-NSD	5.72	0.150	5.12	6.33	WD	MED-DIET	<.001
	MED-DIET	5.73	0.153	5.16	6.31	LFV-HSD	LFV-LSD	0.003
	-	-	-	-	-	LFV-HSD	MED-DIET	0.006
	-	-	-	-	-	LFV-LSD	MED-DIET	1.000
Mathematics	WD	5.10	0.1245	4.85	5.34	WD	LFV-HSD	1.000
	LFV-HSD	5.23	0.0492	5.12	5.34	WD	LFV-LSD	0.151
	LFV-NSD	5.40	0.0406	5.30	5.50	WD	MED-DIET	0.055
	MED-DIET	5.46	0.0522	5.35	5.57	LFV-HSD	LFV-LSD	0.056
	-	-	-	-	-	LFV-HSD	MED-DIET	0.010
	-	-	-	-	-	LFV-LSD	MED-DIET	1.000
Science	WD	5.13	0.1267	4.87	5.39	WD	LFV-HSD	0.142
	LFV-HSD	5.39	0.0778	5.16	5.63	WD	LFV-LSD	0.016
	LFV-NSD	5.47	0.0737	5.22	5.73	WD	MED-DIET	0.001
	MED-DIET	5.57	0.0789	5.33	5.80	LFV-HSD	LFV-LSD	0.846
	-	-	-	-	-	LFV-HSD	MED-DIET	0.033
	-	-	-	-	-	LFV-LSD	MED-DIET	0.665
History	WD	5.27	0.1105	5.05	5.49	WD	LFV-HSD	1.000
	LFV-HSD	5.31	0.0559	5.17	5.46	WD	LFV-LSD	0.237
	LFV-NSD	5.49	0.0507	5.34	5.64	WD	MED-DIET	0.049
	MED-DIET	5.57	0.0574	5.42	5.71	LFV-HSD	LFV-LSD	0.004
	-	-	-	-	-	LFV-HSD	MED-DIET	<.001
	-	-	-	-	-	LFV-LSD	MED-DIET	1.000
PISA	WD	5.11	0.0987	4.92	5.31	WD	LFV-HSD	0.438
	LFV-HSD	5.30	0.0398	5.22	5.39	WD	LFV-LSD	0.013
	LFV-NSD	5.43	0.0331	5.35	5.52	WD	MED-DIET	0.002
	MED-DIET	5.50	0.0420	5.41	5.59	LFV-HSD	LFV-LSD	0.054
	-	-	-	-	-	LFV-HSD	MED-DIET	0.002
	-	-	-	-	-	LFV-LSD	MED-DIET	1.000

Table S5. Post Hoc results of mixed model in comparisons of Mediterranean-style based dietary patterns and academic subjects
